# Understanding Coping Among Spouses of Persons With Alcohol Use Disorder at a General Hospital Psychiatry Unit De-addiction Center: A Cross-Sectional Study

**DOI:** 10.7759/cureus.60317

**Published:** 2024-05-15

**Authors:** Shivani Kanani, Dimple Dadarwala, Harikishan Kanani, Monika Khubchandani, Ramakrishna Yeluri

**Affiliations:** 1 Psychiatry, Shri M.P. Shah Medical College and Guru Gobind Singh Hospital, Jamnagar, IND; 2 Psychiatry, New Civil Hospital, Surat, IND; 3 Pediatric Dentistry, Sharad Pawar Dental College and Hospital, Datta Meghe Institute of Higher Education and Research, Wardha, IND

**Keywords:** mental health, alcohol use disorder, public health, psychiatry, de-addiction

## Abstract

Introduction

Alcoholism is seen as a severe social and health issue. It usually refers to the excessive and unrestrained intake of alcoholic beverages to the point where it becomes harmful to the health, interpersonal connections, and general social functioning of the drinker. The study aims to comprehend coping strategies used by spouses of people suffering from alcoholism and to make significant contributions to the fields of addiction and mental health services.

Methodology

The study was conducted at a General Hospital Psychiatry Unit De-addiction Center from November 2020 to April 2021. Fifty spouses of people with alcohol use disorder (AUD) diagnoses took part in total. Structured questionnaires were used to gather sociodemographic data. The degree of AUD was measured with the Severity of Alcohol Dependence Questionnaire (SADQ), and coping mechanisms were examined with the Questionnaire of Coping Strategies Used by Spouses of Alcoholic Clients.

Results

Out of 120 screened patients, 50 spouses participated in the study. The mean age of the spouses was 33.66 years and 35.08 years for husbands. Sixty percent of the spouses mainly worked in unskilled labor, and the majority (80%) were between the ages of 26 and 35. Based on SADQ scores, about 50% of husbands exhibited severe alcohol dependence. Among the spouses, engaged coping techniques were more common than tolerant or withdrawal coping strategies.

Conclusion

The results highlight the need for specialized therapies and support services to help spouses of people with AUD better manage their stress and improve their general well-being. Comprehending coping mechanisms within this framework can enhance therapy practice and lead to better results for AUD sufferers and their families.

## Introduction

Alcoholism is seen as a serious social and health issue. It usually refers to the excessive and unrestrained intake of alcoholic beverages to the point where it becomes harmful to the health, interpersonal connections, and general social functioning of the drinker. The "alcohol alliance policy" states that there are approximately 62.5 million alcohol consumers in India. India is the world's third-largest market for alcoholic beverages due to its high rates of alcohol consumption [1].

A person with alcohol use disorder (AUD) may experience interpersonal conflicts with others, and if they are married, their relationship with their spouse is often strained the most. The partners of those who suffer from alcoholism often endure severe stress and trauma in their homes. Domestic violence, emotional abuse, financial strain, damage to social reputation, and challenges with sexual intimacy are among the commonly occurring and well-recognized issues faced by partners of individuals with AUD. These difficulties often lead to significant psychological distress for them [2]. Drinking has detrimental social effects, and stressful life events can set off hormonal, behavioral, and psychological reactions that combine to make it hard for a spouse to change. High levels of anxiety, disappointment, neuroticism, and low self-esteem are some of the characteristics that make up the slope [3].

Whenever there is a crisis, people use different modalities of coping. The combined behavioral and psychological tactics that partners of patients with AUD use to master, tolerate, lessen, or limit the stress that comes with their husbands' drinking are known as coping mechanisms. In general, the main objective of this type of coping is believed to be problem- or emotion-focused [1].

The Western context may not be adequate for understanding the same in the Indian context, as the sociocultural background is different. Against this background, this study intends to understand the coping of spouses of patients with AUD [4]. Clinicians' general quality of life, coping mechanisms, and burden can all be improved with these insights. The investigation itself may also impact treatment and outcomes for individuals with AUD.

Aims and objectives

(i) To investigate the coping mechanisms of patients' spouses who have been diagnosed with alcohol consumption disorders. (ii) To find a correlation between coping with sociodemographic detail and the severity of AUD.

## Materials and methods

The study was conducted in the de-addiction center's psychiatric unit at a General Hospital in Surat, Gujarat (India). A cross-sectional observational study design was used to examine the coping strategies of spouses of individuals diagnosed with AUD and explore potential correlations between sociodemographic factors and the severity of AUD from November 2020 to April 2021.

The Medical College and Hospital's Institutional Ethics Committee approved the research. The study's goal was explained to the participants, and both husbands and their spouses provided signed informed consent. The study included all spouses who were between the ages of 20 and 60, had been married for at least five years, and had a husband who met the Diagnostic and Statistical Manual of Mental Disorders, Fifth Edition (DSM-5) criteria for AUD. Husbands with any other psychiatric history, except alcohol and tobacco use disorders, and spouses with other current or previous psychiatric illnesses were not eligible.

Consecutive patients with AUD and their spouses were screened. Couples who fulfilled all the inclusion criteria and gave written consent were included in the study. Sociodemographic details for both males and females were assessed using semi-structured proforma. The severity of alcohol use was assessed by the Severity of Alcohol Dependence Questionnaire (SADQ). The Oxford University "Questionnaire of Coping Strategies Used by Spouses of Alcoholic Clients" was used to evaluate the coping skills of the spouses of patients with AUD.

One of the most often used instruments for determining the extent of alcohol consumption is the SADQ. Physical withdrawal, affective withdrawal, craving and withdrawal relief drinking, typical daily alcohol consumption, and rapidity of reinstatement are the five subscales, each of four items. Every item has a four-point Likert scale attached to it, with the options "rarely" to "nearly always." A score between 0 and 3 corresponds to each item. As a result, the highest possible score is 60, and the lowest is 0. The questionnaire takes between two and five minutes to administer for study. The result is interpreted as 0-7 non-dependent, 8-15 mild dependence, 16-30 moderate dependence, and 31-60 severe dependence. A more recent version of the SADQ (SADQ-C) has been used in this study [5]. Cronbach's alpha reliability coefficient was found to be α=0.914. Item and total score correlation coefficients of the scale items were between 0.309 and 0.730 (p<0.01). The test-retest correlation coefficient of the scale was found to be 0.855 (p<0.01) [6].

The Oxford University "Questionnaire of Coping Strategies Used by Spouses of Alcoholic Clients" was developed to evaluate the coping skills of the spouses of patients with AUD. It measures three basic types of coping: tolerant, engaged, and withdrawal. Scoring: Each item is rated on a four-point Likert scale ranging from 0 to 3, where no=0, occasionally=1, frequently=3, and once or twice=1. To calculate the total score for coping (CQ-TOT), all 30 items are added up. For engaged coping (CQ-E), the scores for the following items are added: 1, 5, 6, 7, 9, 11, 13, 16, 17, 19, 21, 25, 26, 28. For tolerant coping (CQ-T), the scores for the following items are added: 3, 4, 10, 14, 20, 23, 24, 27, 30. For withdrawal coping (CQ-W), the scores for the following items are added: 2, 8, 12, 15, 18, 29. The scores for items 5 and 22 are subtracted. Then, six is added to ensure all CQ-W values are positive [5].

Every case was assessed, examined, and reviewed with a consultant. The results were then determined using the scoring manual provided. The relationship between coping and the severity of alcohol use was discussed and evaluated.

Statistical analysis

Microsoft Excel (Microsoft Corp., Redmond, USA) was used to enter the data, and IBM SPSS Statistics (IBM Corp., Armonk, USA) version 27.0 was used to analyze it. A proportion was utilized to illustrate the fundamental traits of the subjects. Correlation statistics were used for continuous data. Sample t-tests were applied to both continuous and categorical data. The threshold of 'p' less than 0.05 was used to determine statistical significance.

## Results

A total of 120 patients diagnosed with AUD and their spouses were screened in a tertiary care psychiatry setup over a study period of six months. Out of these, 34 had some or other psychiatric disorders, like adjustment disorder, major depressive disorder, or generalized anxiety disorder. About 36 patients didn’t consent to involvement in the study, so 70 out of 120 were not included. Finally, 50 study subjects completed the study in its entirety.

The mean age of the 50 participants (spouses) was 33.66 years. Among this, a maximum of 80% were between 26 and 35 years of age and 6% were up to 25 years of age. Regarding education, it was observed that 48% studied up to primary school and 8% were graduates. Out of this, 18% of spouses were skilled workers, 60% were unskilled workers, and 22% were homemakers (Table 1).

**Table 1:** Demographic details of spouses of persons with alcohol use disorder

Variable	Categories	Frequency (N)	Percentage (%)
Age (in years)	Up to 25	3	6
	26-35	40	80
	36-45	7	14
Education	Illiterate	12	24
	Up to Primary	24	48
	Up to Secondary	10	20
	Graduate	4	8
Occupation	Skilled	9	18
	Unskilled	30	60
	Housewife	11	22

The mean age of the 50 participants (husbands) was 35.08 years. Among this, a maximum of 52% of participants were between 26-35 years of age and only 2% of participants were below 25 years of age. With regards to education, a maximum of 46% of participants were illiterate and 8% were graduates. As far as occupation is concerned, 44% were skilled workers and 56% were unskilled workers. About 50% of husbands were consuming alcohol for 20-25 years and 4% of husbands were using alcohol for less than 10 years (Table 2).

**Table 2:** Demographic details of husbands

Variable	Categories	Frequency (N)	Percentage (%)
Age (in years)	Up to 25	1	2
	26-35	26	52
	36-45	23	46
Education	Illiterate	23	46
	Primary	12	24
	Secondary	11	22
	Graduate	4	8
Occupation	Skilled	22	44
	Unskilled	28	56
Total years of alcohol use	<10	2	4
	10-15	2	4
	15-20	15	30
	20-25	25	50
	>25	6	12
Alcohol-related problem	1) Social problem	39	78
	2) Physical	34	68
	3) Legal	29	58
	4) Risk-taking behavior	16	32
	5) Bodily injury	15	30

SADQ scoring criteria are given in Table 3. Half of the patients in the study (50%) were found to have a severe dependence on the SADQ scale, approximately 40% of patients fell in the moderate dependence category, and only 10% of patients were found to be mild dependence on the SADQ scale (Table 4).

**Table 3:** SADQ scoring criteria SADQ: Severity of Alcohol Dependence Questionnaire

SADQ	Score	Frequency (N)	Percentage (%)
Mild	8-15	5	10
Moderate	16-30	20	40
Severe	31-60	25	50

**Table 4:** Magnitude of severity of alcohol use in husbands

Variables	Score	Frequency (N)	Percentage (%)
Total years of alcohol use	<10	2	4
	10-15	2	4
	15-20	15	30
	20-25	25	50
	>25	6	12
Alcohol-related problem	1) Social problem	39	78
	2) Physical	34	68
	3) Legal	29	58
	4) Risk-taking behaviour	16	32
	5) Bodily injury	15	30

Many spouses, 36 (72%), used financial engaged coping by refusing to give money to their husbands. Around 33 (66%) spouses talked with their husbands to quit alcohol use, and 33 (66%) spouses made rules like forbidding drinking in the house. Arguing was another form of engaged coping that was used by 26 (56%) spouses. Only 10 (20%) of spouses got emotionally attached to their husbands. The results of the tolerant coping rating showed that 30 (60%) of the spouses gave money to their alcoholic partner even though they knew it would be spent on alcohol; 21 (42%) spouses felt helpless to take action, and only five (10%) spouses attempted to maintain the appearance of regularity by pretending everything was fine when it wasn't. The third most regularly used coping approach was withdrawal coping. A majority of spouses, 42 (84%), prioritized the needs of their family members over those of their husbands. Nearly half, 29 (56%), reported using avoidance as a coping strategy (Table 5).

**Table 5:** Coping in spouses

Domains	Frequency (%) No (0)	Frequency (%) Once or twice (1)	Frequency (%) Sometimes (2)	Frequency (%) Often (3)
ENGAGED COPING				
He denied to provide with cash or offer any other financial support.	1(2%)	5(10%)	8(16%)	36(72%)
Sat down together and had an open conversation about addressing his drinking problem.	1(2%)	3(6%)	13(26%)	33(66%)
Engaged in a discussion with him about his drinking, which escalated into an argument.	4(8%)	3(6%)	17(34%)	26(52%)
Pleaded with him regarding his alcohol intake.	2(4%)	5(10%)	12(24%)	31(62%)
Made it very clear to him that his drinking was causing distress and that it needed to change.	5(10%)	5(10%)	16(32%)	24(48%)
Tried to cut down on his drinking by enforcing boundaries, including not allowing him to bring friends who are heavy drinkers home.	2(4%)	1(2%)	14(28%)	33(66%)
Prompted him to swear an oath or abstain from alcohol.	0(0%)	2(4%)	18(36%)	30(60%)
Became moody or emotional with him.	14(28%)	11(22%)	15(30%)	10(20%)
Maintained a close eye on him or closely monitored his actions.	4(8%)	11(22%)	23(46%)	12(24%)
Made it apparent that you wouldn't put up with his drinking excuses or stand by him.	3(6%)	7(14%)	21(42%)	19(38%)
Expressed your expectations for him in terms of what he should do to support the family clearly.	4(8%)	2(4%)	18(36%)	26(52%)
Accused him of being unloving or of failing you.	7(14%)	7(14%)	10(20%)	25(50%)
Sat down with him to assist in organizing his financial circumstances.	0(0%)	12(24%)	18(36%)	19(38%)
Have you looked for his drink, stashed it somewhere, or disposed of it yourself?	3(6%)	7(14%)	23(46%)	16(32%)
TOLERANT COPING				
Did you go above and beyond for him, like clearing the table after a night out or assisting with his bed?	1 (2%)	1 (2%)	5 (10%)	43 (86%)
Given him money even when you suspected it would be used for alcohol.	1(2%)	3 (6%)	16 (32%)	30 (60%)
Felt too frightened to do anything.	3 (6%)	4 (8%)	10 (20%)	33(66%)
Felt too hopeless to do anything.	4 (8%)	10(20%)	15(30%)	21(42%)
Made threats that you had no intention of sustaining.	5(10%)	10(20%)	21(42%)	14(28%)
Became overwhelmed or distressed to the point where you couldn't make any decisions.	2(4%)	4(8%)	10(38%)	25(50%)
Accepted the situation as a part of life that couldn’t be changed.	3(6%)	4(8%)	18(36%)	25(50%)
Did you cover up for him, make excuses, or take the fall when things went wrong because of his drinking?	4(8%)	9(18%)	14(28%)	22(44%)
Attempting to keep things seeming normal, acting as though nothing was wrong, or keeping his real drinking habits a secret from others.	3(6%)	22(44%)	19(38%)	5(10%)
WITHDRAWAL COPING				
Prioritized the needs of other family members over his own.	0(0%)	0(0%)	8(16%)	42(84%)
Did you leave him alone to take care of himself when he was drunk, or did you stay out of his way?	0(0%)	2(4%)	32(64%)	16(32%)
Have you followed your own interests, sought out new hobbies or careers, or increased your involvement in political, religious, athletic, or other organizations?	8(16%)	7(14%)	11(22%)	24(48%)
Avoided him as much as possible because of his drinking.	0(0%)	2(4%)	19(38%)	29(58%)
Moved on with your own affairs or pretended that he wasn't this.	2(4%)	7(14%)	26(52%)	15(30%)
Prioritize yourself occasionally by taking care of yourself or rewarding yourself.	13(26%)	10(20%)	13(26%)	13(26%)

The average tolerant coping score was 17.88 out of 30. Spouses of individuals with AUD demonstrated a more adaptive coping style, with 20 of them scoring above the mean. The mean score for engaged coping was 31.18. A total of 32 spouses of persons with AUD had a score above the mean in engaged coping strategy, and 18 spouses had a score below the mean. The mean score for withdrawal coping was 14.8. A total of 42 spouses of persons with AUD had a better withdrawal coping strategy with a score below the mean, and only eight spouses had a score above the mean (Table 6).

**Table 6:** Individual coping strategy

Individual coping	Mean score	Number of spouses above mean	Number of spouses below mean
Tolerant coping	17.88	20	30
Engaged coping	31.18	18	32
Withdrawn coping	14.8	8	42

As the severity of alcohol use on the SADQ scale increased, the total coping score for spouses also increased. Positive Pearson correlation was calculated to be 0.421, which was statistically significant (p=0.02). Age, family members, and total years of marriage had a negative correlation with the total coping score of spouses with a p-value of 0.304, 0.916, and 0.034, respectively (Table 7).

**Table 7:** Correlation of coping with demographic details of spouses p-value <0.05 is considered statistically significant with a confidence interval of 95%.

Total coping score		
	Pearson correlation	p-value
Age of spouses	-0.148	0.304
Number of family members	-0.015	0.916
Total years of marriage	-0.301	0.034**

The age of the husband and total years of alcohol use had a negative correlation with the total coping score of spouses with a p-value of 0.108 and 0.245, respectively. The income per month had a positive correlation with the total coping score of spouses; this association was statistically not significant with p=0.215 (Table 8).

**Table 8:** Correlation of coping with demographic details of husbands

Total coping score		
	Pearson correlation	p-value
Age of husband	-0.230	0.108
Income per month	0.179	0.215
Total years of alcohol use	-0.167	0.245

## Discussion

Spouses of alcoholic clients frequently deal with a variety of issues. The range of issues spans from minor emotional slights to acts of physical aggression. The current study emphasizes the issues that spouses of patients with alcohol disorders experienced and the different coping mechanisms that they used.

The current study saw three main coping types: withdrawal, tolerance, and engagement. When compared to withdrawal coping (14.8), the mean score for engaging coping (31.18) and tolerated coping (17.88) was higher. While in the West, women tend to use withdrawal coping more often because they are more independent and have access to sufficient community resources to interact with life outside of their alcoholic husband, in Indian contexts, coping strategies are either more engaged or tolerant. In the first few years of a problem, individuals start with tolerant styles, but a desire to improve often results in more engaged coping. After using such techniques for years without seeing any progress, the women are finally forced to use withdrawal coping mechanisms [7]. Earlier studies (Orford et al., 1975; Rao and Kuruvilla, 1992) indicated that coping techniques such as indulgence, shying away from conflict, and assertiveness were widespread, but taking unusual action and acting assertively were less common [8]. This is also contrary to the previous study by Srijana Pandey and Kalpana Shrestha at Nepal Medical College, which shows most spouses used withdrawal coping and then tolerant coping, and very few used coping [3].

As the severity of alcohol use increases in husbands, the coping score in spouses also increases. Using various coping strategies, women learn to live with husbands with AUD. This is consistent with other research that shows a steady increase in all forms of coping in married women based on the severity and frequency of drinking (James and Goldmann, 1971). The spouses gain control, find work, communicate their feelings, and manage the household by implementing a coping strategy. When faced with ongoing stressful situations, humans can cope using behavioral and cognitive techniques.

The current study identified strong and significant associations between certain demographic variables of spouses and their coping strategies. Specifically, educational qualification, employment status, monthly family income, and types of family were found to be significantly associated with coping strategies. These findings are consistent with previous research. For instance, Meenakshi (2003) highlighted significant associations between coping and income, duration of disease, and type of family, while Revathi (2009) also identified a significant association between spouses coping styles and family income [9]. However, some studies have reported contrasting findings. Chandrasekaran and Chitraleka (1998) found no correlation between the educational status of spouses and coping style, and Rao and Kuruvilla (1992) reported no correlation between coping behaviors and variables such as duration of marriage, duration of husband's alcoholism, socioeconomic status, and educational status [8,10]. Orford et al. (2001) evaluated relatives of people with substance use disorders in Mexico and England and discovered that tolerant coping behaviors were associated with higher physical and psychological symptoms in those coping. This suggests that tolerant coping can affect family members. The high incidence of tolerant coping among women in our study suggests poor physical and psychological health, requiring more evaluation and support [11].

Limitations of this study include a relatively small sample size, which may restrict the generalizability of findings to a broader population. The study was conducted within a specific hospital setting, which might not represent coping strategies among spouses in different contexts or regions. Additionally, reliance on self-report measures for assessing coping and other variables could introduce response biases or inaccuracies. Future research could benefit from larger, more diverse samples and objective measures to enhance the validity and applicability of findings related to coping among spouses of individuals with AUD.

## Conclusions

Understanding the coping mechanisms used by spouses of people with AUD is critical for creating focused interventions and support services. By addressing these individuals' specific needs, we can increase treatment effectiveness and overall family functioning and, ultimately, lead to better outcomes for people with AUD and their families. This study adds essential insights to the domains of addiction and mental health services, emphasizing the significance of a comprehensive approach that takes into account not only the individual with AUD but also the significant others affected by the condition. Implementing individualized interventions based on coping techniques can result in more extensive and effective resources for families affected by AUD.
